# Dietary Supplementation with a Combination of Fibrolytic Enzymes and Probiotics Improves Digestibility, Growth Performance, Blood Metabolites, and Economics of Fattening Lambs

**DOI:** 10.3390/ani12040476

**Published:** 2022-02-15

**Authors:** Gamal A. Mousa, Masouda A. Allak, Mohamed G. Shehata, Nesrein M. Hashem, Ola G. A. Hassan

**Affiliations:** 1 Animal Production Department, Faculty of Agriculture, Fayoum University, Fayoum 63514, Egypt; maa23@fayoum.edu.eg (M.A.A.); oga01@fayoum.edu.eg (O.G.A.H.); 2 Department of Food Technology, Arid Lands Cultivation Research Institute, City of Scientific Research and Technological Applications (SRTA-City), New Borg El Arab, Alexandria 21934, Egypt; gamalsng@gmail.com; 3 Department of Animal and Fish Production, Faculty of Agriculture (El-Shatby), Alexandria University, Alexandria 21545, Egypt

**Keywords:** growing lambs, probiotic, fibrolytic enzyme, digestibility, growth, blood serum

## Abstract

**Simple Summary:**

Sheep meat is one of the most important sources of animal protein throughout the world, specifically in arid and semiarid regions. The meat yield of growing lambs can be maximized by improving the function and health of the digestive system, specifically using sheep diets containing ratios high in fibers. Diets rich in fibrous portions cannot be efficiently hydrolyzed by the endogenous enzymes or by the microbes of the rumen. Therefore, the addition of some feed additives that can improve fiber digestion and/or sustain digestive system eubiosis, such as fibrolytic enzymes, probiotics, and yeast, can be a suitable intervention. Fibrolytic enzymes are gaining importance because they improve the nutrient digestibility and performance of animals without affecting the animals’ health. Probiotics (bacteria and/or yeast) are also important feed additives that can support ruminal microbial activity and enhance gut health and ecology through rumen maturity by favoring microbial establishment. In the present study, dietary supplementation with a combination of fibrolytic enzymes and probiotics (Calfo Care^®^) at 0.5, 1, and 2 kg/ton diet of dry matter increased nutrient digestibility, feed intake and feed conversion, daily weight gain, average total weight gain, and improved most blood parameters of lambs. The addition of 1 kg/ton diet of DM resulted in more economic profit compared with other levels.

**Abstract:**

This study was conducted to evaluate the effects of adding different levels of the combination of fibrolytic enzymes and probiotics (a mixture of bacteria and yeast) on the performance of fattening lambs. Thirty-two male Ossimi lambs (weighing 39 ± 0.24 kg) were divided into four groups randomly (eight animals each). The first group (control ration, G1) was fed on a ration of 60% concentrate feed mixture (CFM), 20% Egyptian clover (EC), and 20% wheat straw (WS). The second (G2), third (G3), and fourth (G4) groups were fed a control ration supplemented with Calfo Care^®^ at concentrations of 0.5, 1, and 2 kg/ton diet of dry matter (DM). Results showed that the G2 and G3 rations significantly (*p* ≤ 0.05) increased the DM, organic matter, crude protein, crude fiber, and ether extract digestibility compared with the G1 and G4 rations. Moreover, the G2 and G3 rations increased (*p* ≤ 0.05) the percentages of total digestible nutrients (TDN), starch values (SV), and digestible crude protein (DCP) compared with the G1 and G4 rations. Both the G2 and G3 rations significantly (*p* ≤ 0.05) increased the TDN, SV, and DCP as kg/day or g/kg w^0.75^ and kg or g/100 kg body weight compared with the G1 and G4 rations. Conversely, the G1 ration significantly decreased the feed conversion of DM, TDN, SV, and DCP compared with the experimental groups. Furthermore, the G2, G3, and G4 rations significantly (*p* ≤ 0.05) increased the total weight gain by 25.34%, 52.20%, and 3.79%, respectively, compared with the G1 ration. The G2, G3, and G4 rations also (*p* ≤ 0.05) increased the concentrations of most hematological parameters, including triiodothyronine, total protein, albumin, and glucose, compared with the G1 ration. Finally, the best net profit was recorded with the G3 ration, followed by the G2, G4, and G1 rations.

## 1. Introduction

Sheep meat is one of the most important and acceptable sources of animal protein throughout the world. Due to the continuous increases in the world’s population, there is a rapidly growing demand for such a type of animal protein source, and thus maximizing sheep flocks’ meat production is a crucial need. The meat yield of growing lambs can be maximized by improving the function (mainly nutrient digestion and absorption efficiency) and health (mainly digestive system eubiosis) of the digestive system [1]. Most sheep rations consist of forages with high fibrous content, particularly because they can degrade plant cell wall material with the microorganisms of the rumen and their associated enzymes. However, rumen digestion is not ideal, and commonly a high amount of plant fiber bypasses the digestive tract without being used [2]. In recent years, feed additives, such as fibrolytic enzymes, probiotic bacteria, and yeast culture, have been widely used in improving ruminal fermentation and, consequently, animal performance, particularly in growing animals. Fibrolytic enzymes can be classified on the basis of their specific activity toward cellulase enzymes, which hydrolyze the fiber of plant cell walls to glucose, and convert xylanase enzymes, which play a vital role in the hydrolysis of 1, 4-b-d-xylosidic linkages in xylans—the constituents of hemicellulose, a structural component of plant cell walls—into d-xylose [3]. The use of enzyme-based products with fibrolytic properties in a ruminant’s ration can contribute efficiently to their digesting more fiber in the rumen, leading to an increase in nutrient digestibility [4,5].

Probiotics are becoming increasingly popular as one of the alternatives to antibiotic growth promoters. Probiotics are defined as a live microbial feed supplement that beneficially improves the composition of the rumen/intestinal microbial community in animals [6]. Several studies have investigated the role of *Bacillus* (*B.*), which has the characteristics of a probiotic in enhancing the microbial balance, digestive processes, and immunity of the hosts [6,7,8]. A study conducted on the effect of *B. subtilis* on ruminants demonstrated that *B. subtilis* altered the rumen fermentation pattern of calves and increased the growth performance of animals [7]. Supplementation with yeast also has several benefits in ruminant nutrition, including enhancing nutrient digestibility, increasing volatile fatty acids, reducing ammonia concentration, and stabilizing rumen pH [9]. The most commonly used yeast strain is *Saccharomyces* (*S.*) *cerevisiae*, which can modulate the composition of the microbial ecosystem, increase nutrient digestibility, and enhance weight gain and feed efficiency [10].

Previous studies have reported that the application of probiotics, yeast, and enzymes increased the dry matter intake (DMI) and growth performance of lambs [6], goat kids [11], and beef steers [12], as well as the nutrient digestibility of different animals [5,13]. Based on these facts, a combination of both probiotics and fibrolytic enzymes may benefit growing animals, particularly those fed on fibrous feed stuff, via different actions. Fibrolytic enzymes can improve fiber digestion, and in addition probiotics (bacteria and yeast) can maintain rumen eubiosis and microbial activity, as well as their positive effects on animals’ health and metabolism. Therefore, the present study was conducted to examine the effects of supplementing rations with different levels of a combination of fibrolytic enzymes, probiotics, and yeast on the nutrient digestibility, growth performance, feed conversion, and blood metabolites of Ossimi lambs. Moreover, an economic evaluation of the experimental rations was performed.

## 2. Materials and Methods

### 2.1. Location and Animal Management

This study was performed at the farm of the Faculty of Agriculture from December 2020 to March 2021. All chemical analyses were conducted at the laboratory of the Animal Production Department, Fayoum University, Fayoum, Egypt. The Fayoum Governorate is located less than a hundred kilometers from Cairo. The faculty farm is located in Damou, Fayoum, and its climate is hot dry, with rare rain in the winter (from December to March) and an average daily temperature of 9–23 °C. The experimental protocol and all procedures applied on animals were approved by Fayoum University Institutional Animal Care and Use Committee (FU-IACUC) with proposal code number: AEC2104.

The male Ossimi lambs were healthy and clinically free of internal and external parasites. They were maintained outdoors with shelter during the day and housed in semi-open barns at night. The lambs were maintained under the same environmental and management conditions. Light bulbs were used to provide lighting of 12 h of light per day for each lamb in the trial. Each lamb in the trial was kept in an area of 2 × 1.5 m^2^. The flooring of the lambs’ pens were the softer floors with low thermal conductivity, such as straw.

### 2.2. Experimental Design

A total of 32 male Ossimi lambs (weighing 39 ± 0.24 kg), with an average age of 6 months, were used in this study. The lambs were divided randomly into four experimental groups of eight lambs each based on their live body weight (BW) at allocation. They were fed individually on the experimental rations for 90 days and were adapted to those rations for 14 days before the start of the feeding trial.

The 1st group (control ration, G1) was fed on a total mixture ration consisting of 60% concentrate feed mixture (CFM: 59% yellow corn, 17.5% wheat bran, 7% soybean meal, 7% broken Baladi beans, 7% undecorated cottonseed meal, 1.5% limestone, 0.2% dicalcium phosphate, 0.3% premix, and 0.5% NaCl on a DM basis), 20% Egyptian clover (EC), and 20% wheat straw (WS). The 2nd (G2), 3rd (G3), and 4th (G4) groups were fed a control ration supplemented with Calfo Care^®^ at concentrations of 0.5, 1, and 2 kg/ton diet of dry matter (DM), respectively. Calfo Care^®^ (Reg 4686; Bach 5006) is a commercial product that is manufactured by BIOSEN Chemical Industries for New Vet Care, Egypt. The product contains 2500 IU of cellulase, 1250 IU of xylanase, 4.5 × 10^10^ CFU of B. licheniformis, 5.5 × 10^10^ CFU of B. subtilis, and 2.2 × 10^10^ CFU of S. cerevisiae per kilogram.

The nutritional requirements of lambs were calculated according to the NRC [14]. Rations were offered twice daily in two equal portions at 8.00 a.m. and 4.00 p.m. Feed intake and refusals (if any) were recorded daily, and the DMI was calculated for each animal. Fresh water was available freely throughout the daytime to the animals. The lambs were weighed biweekly in the morning before feeding.

### 2.3. Digestibility Trial

A digestibility trial was conducted at the end of the experiment during which lambs were maintained in individual digestibility cages for 15 consecutive days. The digestibility of nutrients was determined using the acid-insoluble ash technique, as reported by Van Keulen et al. [15]. Daily feces excreted from each lamb were weighed, and 10% of fresh feces was taken and dried at 60 °C for 24 h to determine the DM content of the feces. Composite samples from the daily dried feces for each lamb were mixed, ground, and stored in a refrigerator for subsequent chemical analysis.

Samples of feeds and feces were collected for the chemical analysis. The mixed samples from the daily dried feces for each lamb, which were collected through the digestibility trial, were examined for the percentage of DM, crude protein (CP, using the macro-Kjeldahl method), ether extract (EE), crude fiber (CF), and ash contents according to AOAC analysis methods [16]. The nitrogen-free extract (NFE) was determined using the following formula: NFE = ((organic matter (OM) − (EE + CP + CF)).

The constituents of the plant cell walls, including neutral detergent fiber (NDF), acid detergent fiber (ADF), and acid detergent lignin, were determined in the feeds and feces, as described by Goering and Van Soest [17]. Feeding values, including the total digestible nutrient (TDN), starch value (SV), digestible crude protein (DCP), and digestible energy (DE), were calculated according to the NRC [18]. The calculation of DE was based on digestible compounds using the following formula: DE = ((5.72 × CP) + (9.05 × EE) + (4.38 × CF) + (4.06 × NFE))/100.

### 2.4. Growth Performance Variables

The total weight gain (TWG, kg) was calculated as the difference between the final and initial weights. The average weight (Av. weight) was calculated as an average between the initial and final weights. Moreover, metabolic body size (Av. weight^0.75^) was also calculated.

### 2.5. Blood Sampling and Analysis

Blood samples were collected at days 0, 30, 60, and 90 of the experimental period. All samples were collected before the morning feeding and watering. Approximately 10 mL of fresh blood was withdrawn from the jugular vein of the lambs in all groups. Each blood sample was divided into three aliquots, (1) one with EDTA for measuring hematological variables, (2) one with sodium fluoride and potassium oxalate for analyzing glucose (Glu), and (3) one without any additives for serum separation. Clear blood serum was divided into aliquots across three Eppendorf tubes and stored at −20 °C for further assays.

Hemoglobin (Hb, g/dL) concentration, red blood cell (RBC) count (×10^6^/µL), white blood cell (WBC) count (×10^3^/µL), lymphocytes (%), platelet (PLT) count (×10^3^/µL), and mean corpuscular volume (MCV, fl) were determined using a hematological analyzer (HA-CLINDIAG, China). Blood serum samples were analyzed for total protein (TP, g/dL), albumin (Alb, g/dL), Glu (mg/dL), total cholesterol (CHO, mg/dL), low-density lipoprotein (LDL, mg/dL), high-density lipoprotein (HDL, mg/dL), triglycerides (TGs, mg/dL), total lipids (TLs, mg/dL), urea (mg/dL), creatinine (mg/dL), alanine aminotransferase (ALT, IU/L), and aspartate aminotransferase (AST, IU/l) were determined using commercial test kits (Spectrum Biotechnology, Egypt) and a spectrophotometer (T80 UV/VIS PG instrument Ltd., Lutterworth, UK). Total globulin (Glb, g/dL) was calculated by subtracting the obtained Alb value from the TP value.

Blood serum total triiodothyronine (T_3_, ng/dL) and total thyroxine (T_4_, µg/dL) levels were determined using a radioimmunoassay (RIA) kit (RIA source Immunoassay S.A., Belgium). The sensitivity of this method was 0.3 and 10.63 nmol/L for T_3_ and T_4_, respectively.

### 2.6. Simple Economic Evaluation

Simple financial returns of the tested rations were calculated assuming that the price of 1 kg live BW gain of lambs was EGP 75 (Egyptian pounds). The cost of 1 ton DM of CFM (91.40% DM), EC (15% DM), and WS (95.62% DM) was EGP 5880, 350, and 1400, respectively. The price of 1 kg of the commercial product “Calfo Care^®^” was EGP 50.

### 2.7. Statistical Analysis

For data measured once at a time, a one-way analysis of variance (ANOVA) followed by Duncan’s multiple range test was used [19]. The mathematical model was: Yij = µ + Ti + eij, where Yij is the parameter under analysis, µ is the overall mean, Ti is the effect of treatment, and eij is the experimental error. For repeated measurements (the results of hematobiochemical variables), data were subjected to the statistical analysis using a factorial two-way ANOVA in a randomized complete block design to consider the effect of treatment, time of blood samples collection, and the interaction between the treatment and time. Statistics were calculated using the following mathematical model: Yij = µ + Ti + Pj + TPij + eij, where Yij is the dependent variable in the study, µ is the overall mean, Ti is the effect of treatment (i = 1, 2, 3, 4), Pj is the TIME effect of blood sample collection (j = 1, 2, 3, 4), TPij is the interaction between treatment and time, and eij is the error. All results were expressed as mean and standard error (mean ± SEM). All results were analyzed using IBM SPSS Statistics for Windows, version 22.0 [20].

## 3. Results

### 3.1. Chemical Composition of Feed Ingredients and the Tested Rations

The chemical composition of feed ingredients and the tested rations are shown in Table 1, wherein WS exhibited the greatest levels of ash, CF, NDF, ADF, ADF, hemicellulose, cellulose, and lignin compared with CFM and EC. In contrast, WS showed the least levels of CP and EE compared with other ingredients.

### 3.2. Nutrient Digestibility and Feeding Values

As shown in Table 2, compared with G1 (control), the G3 ration, followed by G2, increased (*p* ≤ 0.05) the nutrient digestibility (the DM, OM, CP, CF, and EE) and feeding values (TDN, SV, and DCP), whereas the G4 ration was not different to G1. There were non-significant increases in DE between all the experimental groups (Table 2).

### 3.3. Lamb Performance

As shown in Table 3, adding Calfo Care^®^ to the G2, G3, and G4 rations increased the TWG by 25.34%, 52.20%, and 3.79%, respectively, compared with the G1 control ration. The greatest (*p* ≤ 0.05) TWG of 20.06 kg was observed in G3, followed by G2, G4, and G1, in which the TWG values were 16.52, 13.68, and 13.18 kg, respectively. The average weight (kg) and metabolic body size were increased (*p* ≤ 0.05) in G3 lambs compared with G1 and G4 lambs, while G2 recorded intermediate values. The daily feed intake of lambs, shown in Table 3, revealed nonsignificant differences between the experimental groups in terms of DMI and DE as kg/day or g/kg w^0.75^ and kg or g/100 kg BW. Both the G2 and G3 rations significantly increased the TDN, SV, and DCP as kg/day or g/kg w^0.75^ and kg or g/100 kg BW compared with the G1 and G4 rations, while G4 did not differ to G1. Compared with G1, the greatest (*p* ≤ 0.05) daily feed conversion of DM, TDN, SV, and DCP was with G3, followed by G2 and G4. Moreover, there were nonsignificant differences in the feed conversions of DE between the experimental groups.

### 3.4. Hematological Parameters

**Figure 1:**
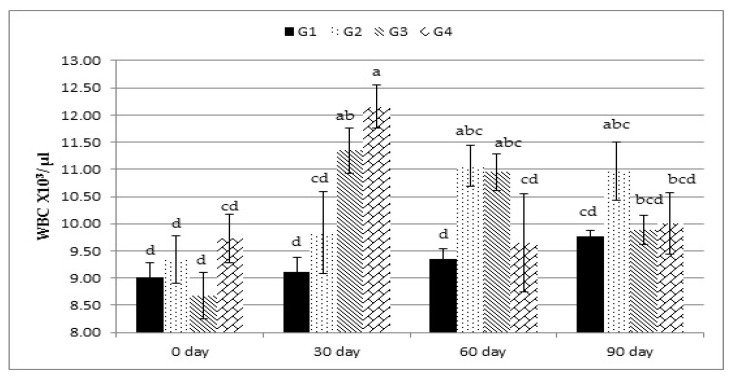
Effect of treatment (G1: 0, G2: 0.5, G3: 1, and G4: 2 kg of Calfo Care/ton DM diet) by time interacion (days 0, 30, 60, and 90) on white blood cell count (WBC) of Ossimi lambs. ^a–d^: Mean values with different superscripts are significantly different (*p* ≤ 0.05).

The overall means of the hemoglobin concentration, RBC count, WBC count, and lymphocyte percentage were significantly (*p* < 0.01) greater in all the Calfo Care^®^ supplemented lambs, and no treatment-by-time interaction effect was observed for all of these variables. Neither treatment nor treatment-by-time interaction affected MCV and PLT (Table 4). There was an increase in WBC count in the lambs fed on the rations supplemented with different levels of Calfo Care^®^. Treatment-by-time interaction analysis revealed that these increases in WBC count in the experimental groups started at 30, 60, and 90 days compared with day 0 (Figure 1).

#### 3.4.1. Blood Biochemical Attributes

Results revealed that the rations supplemented with different levels of Calfo Care^®^ significantly increased the blood serum TP, Alb, and Glu concentrations compared with the unsupplemented ration (G1) (Table 5). The increases in TP were 5.76%, 7.06%, and 7.35% in G2, G3, and G4, respectively, compared with G1. Furthermore, nonsignificant differences were detected in Glb concentrations between all the experimental groups (Table 5).

#### 3.4.2. Fat Profile

**Figure 2:**
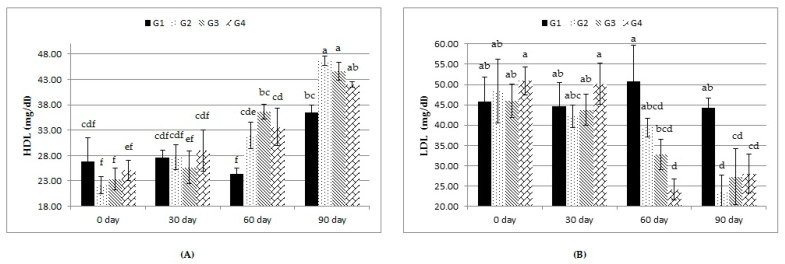
Effect of treatment (G1: 0, G2: 0.5, G3: 1, and G4: 2 kg of Calfo Care/ton DM diet) by time interacion (days 0, 30, 60, and 90) on blood serum levels of high-density lipoprotein (HDL, (**A**)) and low-density lipoprotein (LDL, (**B**)) of Ossimi lambs. ^a–f^: Mean values with different superscripts are significantly different (*p* ≤ 0.05).

As shown in Table 6, neither treatment nor treatment-by-time interaction affected concentrations of blood serum TLs, CHO, and TGs. LDL showed lesser (*p* ≤ 0.05) values with the G2, G3, and G4 rations than with the G1 ration. Moreover, there was a nonsignificant increase in the concentration of HDL in all the experimental groups. All treatments significantly increased the overall mean of blood serum HDL and decreased blood serum LDL compared to the control. The treatment-by-time interaction revealed that these changes were significant at days 60 and 90 days compared to those at 0 and 30 days (Figure 2).

#### 3.4.3. Blood Serum Hormones

As shown in Table 7, the rations supplemented with different levels of Calfo Care^®^ significantly increased the blood serum T_3_ concentration compared with the unsupplemented ration (G1). However, there was a nonsignificant increase in T_4_ concentration in G2, G3, and G4 compared to that in G1.

#### 3.4.4. Kidney and Liver Functions

Adding different levels of Calfo Care^®^ to the lambs’ rations (G2, G3, and G3) decreased (*p* ≤ 0.05) the blood serum urea concentration compared with G1 (Table 7). Moreover, nonsignificant differences were observed in urea concentrations between different periods (Table 7). Nonsignificant differences were also detected in creatinine concentrations between G2, G3, and G4 and G1. Furthermore, there were no significant differences in serum AST and ALT levels between all the experimental groups (Table 7).

### 3.5. A Simple Economic Evaluation of the Tested Rations

Adding Calfo Care^®^ to the G2, G3, and G4 rations increased the net profit by 91.89%, 192.14%, and 9.38%, respectively, compared to that with the G1 ration. The best net profit (EGP/head/90 day) was EGP 763.76 with the G3 ration, followed by the G2, G4, and G1 rations with which the values were EGP 501.67, 285.97, and 261.44, respectively (Table 8).

## 4. Discussion

An adequate nutritional plane is essential for the growth and development of an animal. Not only does an animal require an optimal amount of feed, but it is also crucial to improve the digestibility of feedstuff for maximizing growth [21]. Growing animals may exhibit different metabolic and digestive disturbances that might affect their performance and health. In this study, we aimed to use a combination of both fibrolytic enzymes and probiotics to improve the growth performance and health of fattening lambs. This combination is hypothesized to be beneficial for growing lambs via different actions, mainly by improving fiber digestion due to the action of specific fibrolytic enzymes, and by maintaining rumen eubiosis and microbial activity due to the action of probiotics (bacteria and yeast), as well as the positive effects on animals’ health and metabolism.

In the present study, the use of Calfo Care^®^ (a combination of fibrolytic enzymes and probiotics) increased values of TDN, SV, DCP and DE. This may be due to the increasing digestibility of nutrients, which may be attributed to the accumulation of large amounts of readily fermentable carbohydrates liberated by the action of the fibrolytic enzymes and probiotics in the lambs’ rations [4,5,13]. The improvement of feed intake (TDN, SV and DCP), feed conversion (DM, TDN, SV, and DCP), DWG, TWG, and economic evaluation in the present study may be attributed to the increased nutrient digestibility caused by the addition of the combination.

In fact, throughout our study, the role of each individual component in the Calfo Care^®^ supplement cannot be identified; however, a synergetic effect between all the components can be inferred. The improvement in most nutrient digestibility parameters in this study could be explained on the basis that dietary supplementation with cellulase, xylanase, *B. licheniformis*, *B. subtilis*, and *S. cerevisiae* may enhance the ruminal microbial activity and communities, thus increasing the gut health and ecology through rumen maturity by favoring microbial establishment, increasing the fiber digestion of feedstuff, reducing the fluid viscosity and ruminal ammonia, and improving the concentration of volatile fatty acids in the rumen [8]. Bacterial probiotics, such as *B. licheniformis* and *B. subtilis*, have been used as therapeutic supplements in farm animals to decrease morbidity and mortality [22], improve feeding behavior, and increase production (meat and milk) yield [6]. There are at least two proposed mechanisms by which probiotics can combat unwanted microorganisms, including with the production of inhibitory compounds and/or direct cell-to-cell interactions [23]. Probiotics produce antimicrobial compounds, such as organic acids, hydrogen peroxide, bacteriocins, and bio surfactants, all of which can inhibit the growth of pathogenic microorganisms [24]. Moreover, yeast (*S. cerevisiae*), as a feed additive for ruminants, provides organic acids and vitamins to stimulate the growth of lactic acid bacteria, which improve rumen metabolism by stabilizing the rumen pH, increasing the yield of cellulolytic bacteria, and improving anaerobiosis by scavenging the oxygen available in the rumen, as well as improving microbial protein synthesis and fiber digestibility [25]. Furthermore *S. cerevisiae* reduces the redox potential that creates better conditions for the growth of strict anaerobic microorganisms, and produces specific factors, e.g., vitamin B12 or branched-chain fatty acids, that may stimulate the synthesis of microbial biomass in the rumen [26]. Moreover, adding fibrolytic enzymes, such as cellulase and xylanase, to ruminants’ feeds can reduce the feed viscosity, which increases the absorption of nutrients, and liberates nutrients either through the hydrolysis of non-biodegradable fibers or by liberating nutrients blocked by these fibers [2,5].

Regardless of the positive effects of Calfo Care^®^ on nutrient digestibility and feeding values, it is important to note that the G4 ration (the greatest level of the product) produced negative effects on nutrient digestibility, feeding values, and TWG compared with those obtained with the G2 and G3 rations. This may be attributed to the negative feedback action of the high levels of fibrolytic enzymes. This feedback mechanism occurs when enzyme action is inhibited by the production of a critical concentration of a product of the enzyme–substrate interaction. For instance, the fermentation of sugars produced by cell wall hydrolysis may reduce ruminal pH to levels that inhibit the digestion of the cell wall [27].

In this study, the addition of Calfo Care^®^ to the lambs’ rations improved the hematological and immunological variables, indicating the improved health status of the supplemented lambs. Such effects may be related to the increased synthesis of vitamin B12 by yeast cells and/or improved iron salt absorption by the small intestine, resulting in better hematopoiesis [28]. WBCs are a major component of the body’s immune system and are extremely important in defending the body against infections. The results of WBC count were consistent with those obtained by Milewski and Sobiech [29], who found that yeast-supplemented lambs had a greater WBC count that participated in increasing lymphocyte percentages in the leukogram [30]. In this study, lambs supplemented with different levels of Calfo Care^®^ also showed better blood serum protein and lipid profiles and energy status (Glu levels). The supplementations used in our study improved the blood serum TP. This effect is expected, as protein digestibility was improved in the treated groups. In fact, probiotics can synthesize protease enzymes and, thus, provide some specific amino acids that can boost microbial protein synthesis [30]. In addition, the increases in blood Alb levels may be associated with improved nitrogen absorption driven by adding yeast to the animals’ rations [31]. These findings are in agreement with those of El-Shaer [31] and El-Ashry et al. [32] in sheep, and Abu El-Ella and Kommonna [33] in goats. Treatment with Calfo Care^®^ improved the energy status of growing lambs as indicated by the serum Glu levels. This improvement can be related to improved gluconeogenesis. Previous studies have demonstrated that probiotic supplementation can improve gluconeogenesis by increasing propionate concentrations, which is the primary precursor of Glu, with a decisive influence on the Glu blood concentration in small ruminants [34]. Regarding the lipid profile, our results are in agreement with those obtained by Abas et al. [35], Chiofalo et al. [36], and Baiomy [37], who found that the concentrations of TLs, TGs, and LDL were decreased in kids or lambs that received probiotic supplementation. The reduction in CHO concentration in this study may be due to the inhibition of CHO synthesis or the direct assimilation of CHO [38]. There are two proposed mechanisms for the low circulating CHO concentrations in animals fed with probiotics; specifically, (1) the simultaneous sediment of CHO and the deconjugation of bile acids, and (2) the increase in the degradation of CHO across the gastrointestinal tract [39].

Thyroid hormones exert an important function in the regulation of TGs, CHO metabolism, lipoprotein homeostasis, and the induction of the genes involved in glycolysis and gluconeogenesis [40]. The increases in T_3_ and T_4_ concentrations in the present study may be due to the probiotics’ action on the thyroid-stimulating hormone-releasing hormone (TSH-RH) in the hypothalamus, thus stimulating the release of TSH from the thyrotrophic cells of the anterior pituitary [40].

Urea is the primary end product of nitrogen metabolism in ruminants. It is synthesized in the liver and extracted in the glomeruli. The lesser serum urea concentration in the present study may be because of high protein utilization by lambs supplemented with Calfo Care^®^, which may have been associated with the use of urea for protein synthesis in the hepatic pathway because of a compensation for low protein absorption. These results are consistent with those obtained by Doležal et al. [41], who found lower concentrations of serum urea nitrogen in cows in response to yeast culture supplementation. In contrast, the serum AST and ALT concentrations estimated in the present study were within the normal range, suggesting that the commercial Calfo Care^®^ is safe for the physiological and health status of all experimental groups.

## 5. Conclusions

The rations (G2 and G3) supplemented with Calfo Care^®^ at levels of 0.5 and 1 kg/ton diet of DM increased the nutrient digestibility, the feed intake of TDN, SV, and DCP, feed conversion, DWG, and TWG of Ossimi lambs compared with the baseline (control) and the G4 ration (2 kg/ton DM diet). Calfo Care^®^ supplementation improved the hematological and biochemical parameters of the experimental Ossimi lambs, with protective effects on renal function and positive effects on energy metabolism, and all mean values of blood serum parameters were within the normal range. Adding Calfo Care^®^ to the G2, G3, and G4 rations increased the net profit by 91.89%, 192.14%, and 9.38%, respectively, compared with the G1 ration. Within the context of this study, supplementing Ossimi growing lambs with Calfo Care^®^ at the level of 1 kg/ton DM diet can improve the growth performance and health status of animals with adequate economic revenue.

## Figures and Tables

**Table 1:** Chemical composition of feed ingredients (on % dry matter basis).

Item	CFM	EC	WS	TMR
Organic matter	93.76	82.75	81.41	89.08
Crude protein	16.83	17.29	2.94	14.14
Ether extract	2.86	3.68	0.92	2.64
Crude fiber	4.21	26.00	33.89	14.50
Nitrogen free extract	69.86	35.78	43.66	57.80
Ash	6.24	17.25	18.59	10.92
Neutral detergent fiber	17.56	46.75	69.80	33.85
Acid detergent fiber	6.34	35.85	53.50	21.67
Acid detergent lignin	1.38	8.70	18.45	6.26
Hemicellulose	11.22	10.90	16.30	12.17
Cellulose	4.96	27.15	35.05	15.42
Lignin	0.62	8.75	15.66	5.25

Hemicellulose = NDF − ADF, cellulose = ADF − ADL, CFM: concentrate feed mixture, EC: Egyptian clover, WS: wheat straw, TMR: total mixed ration.

**Table 2:** Effect of dietary supplementation with different levels (G1: 0, G2: 0.5, G3: 1, and G4: 2 kg/ton DM diet) of Calfo Care^®^ on nutrient digestibility and feeding values of lambs.

	Experimental Groups					
Items	G1	G2	G3	G4	±SEM	*p* Value
Nutrient digestibility (%)						
DM	57.41 ^c^	62.79 ^b^	65.39 ^a^	58.55 ^c^	1.27	0.031
OM	62.45 ^c^	66.67 ^b^	68.80 ^a^	63.88 ^c^	1.16	0.025
CP	57.41 ^c^	63.44 ^b^	65.24 ^a^	56.62 ^c^	1.09	0.001
CF	57.51 ^c^	62.74 ^b^	66.70 ^a^	58.90 ^c^	1.14	0.027
EE	63.24 ^b^	75.57 ^a^	77.08 ^a^	59.51 ^b^	2.59	0.011
NFE	63.44	67.50	68.70	64.62	1.38	0.072
Feeding values						
TDN (%)	57.37 ^b^	62.67 ^a^	64.07 ^a^	57.48 ^b^	0.88	0.002
SV (%)	51.32 ^b^	56.52 ^a^	58.47 ^a^	51.43 ^b^	0.45	0.034
DCP (%)	8.32 ^b^	9.20 ^a^	9.46 ^a^	8.21 ^b^	0.13	0.042
DE ( Mcal/kg)	2.49	2.73	2.79	2.49	0.09	0.081

^a–c^: Means in the same row with different superscripts are significantly different (*p* ≤ 0.05). Each value is a mean of eight animals. DM: dry matter, OM: organic matter, CP: crude protein, CF: crude fiber, EE: ether extract, NFE: nitrogen-free extract, TDN: total digestible nutrient, SV: starch value, DCP: digestible crude protein, DE: digestible energy.

**Table 3:** Effect of dietary supplementation with different levels (G1: 0, G2: 0.5, G3: 1, and G4: 2 kg/ton DM diet) of Calfo Care^®^ on total weight gain, feed intake, and feed conversion of lambs.

Items	Experimental Groups				±SEM	*p* Value
	G1	G2	G3	G4		
Av. initial weight (kg/head)	39.28	39.36	39.12	39.16	0.73	0.391
Av. final weight (kg/head)	52.46 ^b^	55.88 ^ab^	59.18 ^a^	52.84 ^b^	1.13	0.043
Total weight gain (kg/head)	13.18 ^c^	16.52 ^b^	20.06 ^a^	13.68 ^c^	0.75	0.029
Daily weight gain(g/head)	146.44 ^c^	183.55 ^b^	222.89 ^a^	152.00 ^c^	0.07	0.018
Av. weight (kg)	45.87 ^b^	47.62 ^ab^	49.15 ^a^	46.00 ^b^	0.19	0.044
Metabolic body size	17.63 ^b^	18.13 ^ab^	18.56 ^a^	17.66 ^b^	0.09	0.041
Av. daily DMI						
DMI (kg/head)	1.44	1.45	1.44	1.43	0.11	0.259
DMI (g/kg w^0.75^)	81.68	79.99	81.53	77.05	0.14	0.169
DMI (kg/100 kg BW)	3.14	3.04	3.13	3.11	0.09	0.103
Av. daily TDNI						
TDN (kg/head)	0.81 ^b^	0.91 ^a^	0.92 ^a^	0.82 ^b^	0.08	0.040
TDN (g/kg w^0.75^)	45.94 ^b^	50.13 ^a^	49.71 ^a^	46.54 ^b^	0.06	0.035
TDN (kg/100 kg BW)	1.77 ^b^	1.91 ^a^	1.88 ^a^	1.79 ^b^	4.30	0.043
Av. daily SVI						
SV (kg/head)	0.74 ^b^	0.82 ^a^	0.84 ^a^	0.74 ^b^	0.03	0.018
SV (g/kg w ^0.75^)	41.92 ^b^	45.21 ^a^	45.37 ^a^	41.65 ^b^	0.15	0.027
SV (kg/100 kg BW)	111.88 ^b^	118.69 ^a^	118.96 ^a^	111.80 ^b^	1.03	0.015
Av. daily DCPI						
DCP (g/head)	119.81 ^b^	133.40 ^a^	136.22 ^a^	117.40 ^b^	0.08	0.001
DCP (g/kg w^0.75^)	6.80 ^b^	7.36 ^a^	7.34 ^a^	6.65 ^b^	0.11	0.002
DCP (g/100 kg BW)	261.19 ^b^	280.13 ^a^	277.16 ^a^	255.22 ^b^	2.07	0.002
Av. daily DEI						
DE (Mcal/day)	3.59	3.96	4.02	3.56	0.14	0.149
DE (Mcal/kg w^0.75^)	0.203	0.218	0.216	0.202	0.17	0.107
DE (Mcal/100 kg BW)	7.817	8.313	8.174	7.741	0.21	0.183
Feed conversion						
DM (kg feed/kg gain)	9.83 ^a^	7.90 ^c^	6.46 ^d^	9.41 ^b^	0.31	0.033
TDN (kg feed/kg gain)	5.53 ^a^	4.95 ^c^	4.14 ^d^	5.41 ^b^	0.13	0.029
SV (kg feed/kg gain)	5.05 ^a^	4.47 ^c^	3.78 ^d^	4.84 ^b^	0.08	0.004
DCP (g feed/g gain)	0.818 ^a^	0.546 ^c^	0.460 ^d^	0.580 ^b^	0.01	0.002
DE (Mcal feed/kg gain)	24.484	21.566	18.03	23.43	0.31	0.091

^a–d^: Mean values in the same row with different superscripts are significantly different (*p* ≤ 0.05). Each value is a mean of eight animals. BW: body weight, Av.: average, DMI: dry matter intake, TDNI: total digestible nutrient intake, SVI: starch value intake, DCPI: digestible crude protein intake, DEI: digestible energy intake.

**Table 4:** Serum hematological parameters of Ossimi lambs supplementated with different levels (G1: 0, G2: 0.5, G3: 1, and G4: 2 kg/ton DM diet) of Calfo Care^®^.

Items	Experimental Groups				±SEM	*p* Value		
	G1	G2	G3	G4		T	P	T × P
Hb (g/dL)	10.11 ^c^	10.68 ^ab^	10.98 ^a^	10.48 ^bc^	0.16	0.001	0.108	0.451
RBCs (×10^6^/µL)	10.50 ^c^	11.50 ^ab^	11.72 ^a^	11.33 ^b^	0.12	<0.001	0.085	0.149
MCV (fl)	39.85	39.87	39.94	39.84	0.59	0.436	0.131	0.463
WBCs (×10^3^/µL)	9.31 ^b^	10.30 ^a^	10.22 ^a^	10.36 ^a^	0.23	0.003	<0.001	0.001
Lymphocytes (%)	62.78 ^b^	69.28 ^a^	69.18 ^a^	69.46 ^a^	1.02	<0.001	0.006	0.442
Platelet (×10^3^/µL)	358.75	254.29	346.46	293.79	32.04	0.050	0.335	0.207

^a–c^: Mean values in the same row with different superscripts are significantly different (*p* ≤ 0.05). Each value is a mean of eight animals. Hb: hemoglobin, RBCs: red blood cells, WBCs: white blood cells, MCV: mean corpuscular volume, T: treatment, P: period/time of sample collection, T × P: interaction between treatments and periods.

**Table 5:** Serum proteins profile and glucose of Ossimi lambs supplemented with different levels (G1: 0, G2: 0.5, G3: 1, and G4: 2 kg/ton DM diet) of Calfo Care^®^.

Items	Experimental Groups				±SEM	*p* Value		
	G1	G2	G3	G4		T	P	T × P
TP (g/dL)	6.94 ^b^	7.34 ^a^	7.43 ^a^	7.45 ^a^	0.13	0.042	0.022	0.099
Alb (g/dL)	3.48 ^b^	3.82 ^a^	3.89 ^a^	3.84 ^a^	0.06	<0.001	<0.011	0.729
Glb (g/dL)	3.47	3.53	3.54	3.61	0.14	0.925	0.095	0.148
Glu (mg/dL)	66.01 ^b^	73.31 ^a^	73.02 ^a^	73.09 ^a^	1.42	<0.002	<0.001	0.164

^a–b^: Mean values in the same row with different superscripts are significantly different (*p* ≤ 0.05). Each value is a mean of eight animals. TP: total protein, Alb: albumin, Glb: total globulin, Glu: glucose, T: treatment, P: period/time of sample collection, T × P: interaction between treatments and periods.

**Table 6:** Peripheral blood of serum fat profile of Ossimi lambs supplemented with different levels (G1: 0, G2: 0.5, G3: 1, and G4: 2 kg/ton DM diet) of Calfo Care^®^.

Items (mg/dL)	Experimental Groups				±SEM	*p* Value		
	G1	G2	G3	G4		T	P	T × P
TGs	84.67	78.83	77.33	79.88	3.35	0.454	0.878	0.932
CHO	92.17	86.25	85.50	86.75	2.58	0.266	0.486	0.356
HDL	28.34 ^b^	32.12 ^a^	32.58 ^a^	32.44 ^a^	1.23	0.085	<0.001	0.024
LDL	46.39 ^a^	38.37 ^b^	37.45 ^b^	38.33 ^b^	2.52	0.049	<0.001	0.048
TLs	269.00	251.33	248.33	253.38	6.51	0.122	0.479	0.451

^a–b^: Mean values in the same row with different superscripts are significantly different (*p* ≤ 0.05). Each value is a mean of eight animals. TGs: triglycerides, CHO: total cholesterol, HDL: high-density lipoprotein, LDL: low-density lipoprotein, TLs: total lipids, T: treatment, P: period/time of sample collection, T × P: interaction between treatments and periods.

**Table 7:** Peripheral blood levels of serum hormones and kidney and liver function of Ossimi lambs supplemented with different levels (G1: 0, G2: 0.5, G3: 1, and G4: 2 kg/ton DM diet) of Calfo Care^®^.

Items	Experimental Groups				±SEM	*p* Value		
	G1	G2	G3	G4		T	P	T × P
T_3_ (ng/dL)	122.83 ^b^	136.74 ^a^	134.13 ^a^	133.99 ^a^	2.35	0.002	0.000	0.006
T_4_ (µg/dL)	8.92	9.35	9.26	9.28	0.28	0.730	0.000	0.069
Urea(mg/dL)	33.96 ^a^	29.25 ^b^	28.58 ^b^	28.95 ^b^	1.44	0.039	0.220	0.801
Creat(mg/dL)	0.80	0.83	0.85	0.85	0.04	0.767	0.012	0.169
Liver function								
ALT (IU/L)	17.00	16.92	16.83	16.88	1.09	1.00	0.912	0.985
AST (IU/L)	88.63	95.08	88.75	90.17	2.72	0.266	0.079	0.767

^a–b^: Mean values in the same row with different superscripts are significantly different (*p* ≤ 0.05). Each value is a mean of eight animals. T_3_: total triiodothyronine, T_4_: total thyroxine, Creat: creatinine, ALT: alanine aminotransferase, AST: aspartate aminotransferase, T: treatment, P: period/time of sample collection, T × P: interaction between treatments and periods.

**Table 8:** Averages of economic evaluation of lambs supplemented with different levels (G1: 0, G2: 0.5, G3: 1, and G4: 2 kg/ton DM diet) of Calfo Care^®^ during testing periods.

Items	Experimental Groups			
	G1	G2	G3	G4
TWG (kg/head/90 day)	13.18	16.52	20.06	13.68
DMI (kg/head/90 day)	129.60	130.50	130.50	128.70
Price of one kg DM of the ration (EGP)	5.61	5.65	5.68	5.75
Cost of feed intake (EGP/head/90 day)	727.06	737.33	741.24	740.03
Total profit(EGP) *	988.50	1239	1505	1026
Net profit (EGP) **	261.44	501.67	763.76	285.97

* Total profit, EGP (Egyptian pound) = total weight gain (kg/head/90 days) × EGP 75 (the price of one kg of live body weight). ** Net profit (EGP/head/90 day) = total revenue (EGP/head/90 day) − cost of feed consumed (EGP/head/90 day).

## Data Availability

Data are confidential, and its availability depends on the authors’ permission.

## References

[1] Food and Agriculture Organization of the United Nations, Statistics Division, FAOSTAT. http://www.fao.org/faostat/ar/#data/QA.

[2] Azzaz H.H., Abd El Tawab A.M., Khattab M.S.A., Szumacher-Strabel M., Cie’slak A., Murad H.A., Kiełbowicz M., El-Sherbiny M. (2021). Effect of Cellulase Enzyme Produced from Penicillium chrysogenum on the Milk Production, Composition, Amino Acid, and Fatty Acid Profiles of Egyptian Buffaloes Fed a High-Forage Diet. Animals.

[3] Sujani S., Seresinhe R.T. (2015). Exogenous Enzymes in Ruminant Nutrition: A Review. Asian J. Anim. Sci..

[4] El-Garhy G.M., Abd El-Mola A.M., Azzaz H.H., Mousa G.A. (2020). Influence of using pectinase enzymes in the ration on nutrient digestibility, blood chemistry, milk composition and economics of lactating buffaloes. J. Anim. Health Prod..

[5] Mousa G.A., Allak M.A., Hassan O.G.A. (2022). Influence of fibrolytic enzymes supplementation on lactation performance of Ossimi ewes. Adv. Anim. Vet. Sci..

[6] Jia P., Cui K., Ma T., Wan F., Wang W., Yang D., Wang Y., Gue B., Zhao L., Diao Q. (2018). Influence of dietary supplementation with *Bacillus licheniformis* and *Saccharomyces cerevisiae* as alternatives to monensin on growth performance, antioxidant, immunity, ruminal fermentation and microbial diversity of fattening lambs. Sci. Rep..

[7] Hashem N.M., Hosny N.S., El-Desoky N.I., Shehata M.G. (2021). Effect of Nanoencapsulated Alginate-Synbiotic on Gut Microflora Balance, Immunity, and Growth Performance of Growing Rabbits. Polymers.

[8] Jiang B., Wang T., Zhou Y., Li F. (2020). Effects of enzyme + bacteria treatment on growth performance, rumen bacterial diversity, KEGG pathways, and the CAZy spectrum of Tan sheep. Bioengineered.

[9] Wang Z., He Z., Beauchemin K.A., Tang S., Zhou C., Han X., Wang M., Kan J., Odongo N.E., Tan Z. (2016). Comparison of two live Bacillus species as feed additives for improving in vitro fermentation of cereal straws. Anim. Sci. J..

[10] Stella A.V., Paratte R., Valnegri L., Cigalino G., Soncini G., Chevaux E., Dell’Orto V., Savoini G. (2007). Effect of administration of live Saccharomyces cerevisiae on milk production, milk composition, blood metabolites, and faecal flora in early lactating dairy goats. Small Rumin. Res..

[11] Salvedia C.B., Supungco E.P. (2017). Effect of probiotic supplementation on weight gain, blood biochemical and hematological indices of crossbred dairy goat kids. Glob. Adv. Res. J. Agric. Sci..

[12] Salem A.Z.M., Gado H.M., Colombatto D., Elghandour M.M.Y. (2013). Effects of exogenous enzymes on nutrient digestibility, ruminal fermentation and growth performance in beef steers. Livest. Sci..

[13] Aboul-Fotouh G.E., El-Garhy G.M., Abd El-Mola A.M., Mousa G.A., Azzaz H.H. (2017). Effect of using some fibrolytic enzymes in the ration on lactating goats performance. Egypt. J. Nut. Feeds..

[14] National Research Council (NRC) (2007). Nutrient Requirement of Small Ruminants: Sheep, Goats, Cervids, and New World Camelid.

[15] Van Keulen J.V., Young B.A. (1977). Evaluation of acid insoluble ash as a natural marker in ruminant digestibility studies. J. Anim. Sci..

[16] AOAC (2009). Official Methods of Analysis of AOAC International.

[17] Goering H.K., Van Soest P.J. (1970). Forage Fiber Analysis (Apparatus, Reagents, Procedures and Some Applications). Agricultural Handbook.

[18] NRC (2001). Nutrient Requirement of Small Ruminants.

[19] Duncan D.B. (1955). Multiple range and multiple F tests. Biometrics.

[20] IBM Corp Released (2013). IBM SPSS Statistics for Windows.

[21] Owens F.N., Dubeski P., Hanson C. (1993). Factors that alter the growth and development of ruminants. J. Anim. Sci..

[22] Rai V., Yadav B., Lakhani G. (2013). Applications of probiotic and prebiotic in animals production: A review. Environ. Ecol..

[23] Jonkers D.M. (2016). Microbial perturbations and modulation in conditions associated with malnutrition and malabsorption. Best Pract. Res. Clin. Gastroenterol..

[24] Chaucheyras-Durand F., Durand H. (2010). Probiotics in animal nutrition and health. Benef. Microbes.

[25] Bomba A., Nemcova R., Gancarcikova S., Herich R., Guba P., Mudronova D. (2002). Improvement of the probiotic effect of micro-organisms by their combination with maltodextrins, fructo-oligosacharides and polyunsaturated fatty acids. Br. J. Nutr..

[26] Chaucheyras-Durand F., Walker N.D., Bach A. (2008). Effects of active dry yeasts on the rumen microbial ecosystem: Past, present and future. Anim. Feed Sci. Technol..

[27] Beauchemin K.A., Rode L.M., Maekawa M., Morgavi D.P., Kampen R. (2000). Evaluation of anon-starch polysaccharidase feed enzyme in dairy cow diets. J. Dairy Sci..

[28] LaFleur-Brooks M., LaFleur-Brooks D. (2008). Exploring Medical Language: A Student-Directed Approach.

[29] Milewski S., Sobiech P. (2009). Effect of dietary supplementation with Saccharomyces cerevisiae dried yeast on milk yield, blood biochemical and hematological indices in ewes. Bull. Vet. Inst. Pulawy.

[30] Talha M.H., Moawd R.I., Abu El-Ella A.A., Zaza G.H. (2009). Effect of some feed additive on rearing calves from birth till weaning:1- Productive performance and some blood parameters. J. Anim. Poult. Prod..

[31] El-Shaer E.K.H.I. (2003). Nutritional Studies in Ruminants. Effect of Yeast Culture Supplementation and Concentrate: Roughage Ratio on Performance of Growing Lambs. Ph.D. Thesis.

[32] El-Ashry M.A., Fayed A.M., Youssef K.M., Salem F.A., Hend A.A. (2003). Effect of feeding flavomycin or yeastas feed supplement on lamb performance in Sinai. Egypt. J. Nutr. Feed..

[33] Abu El-Ella A.A., Kommonna O.F. (2013). Reproductive performance and blood constituents of Damascus goats as affected by yeast culture supplementation. Egypt. J. Sheep Goat Sci..

[34] Sayed A.S. (2003). Studies on the influence of pronifer as a probiotic on the clinical, hematological and biochemical status of the goat’s kids. Assiut Vet. Med. J..

[35] Abas I., Kutay H.C., Kahraman R.S., Toker N.Y., Ozcelik D., Ates F., Kacakci A. (2007). Effects of organic acid and bacterial direct-fed microbial on fattening performance of Kivircik-Male yearling lambs. Pak. J. Nutr..

[36] Chiofalo V., Liotta L., Chiofalo B. (2004). Effects of the administration of Lactobacilli on body growth and on the metabolic profile in growing Maltese goat kids. Reprod. Nutr. Dev..

[37] Baiomy A.A. (2011). Influence of live yeast culture on milk production, composition and some blood metabolites of Ossimi ewes during the milking period. Am. J. Biochem. Mol. Biol..

[38] Zarate G., Chaia A.P., Oliver G. (2002). Some characteristics of practical relevance of the β-Galactosidase from potential probiotic strains of Propionibacterium acidipropionici. Anaerobe.

[39] Yen P.M., Feng X., Flamant F., Chen Y., Walker R.L., Chassande O., Samarut J., Refetoff S., Meltzer P.S. (2003). Effects of ligand and thyroid hormone receptor isoforms on hepatic gene expression profiles of thyroid hormone receptor knockout mice. EMBO Rep..

[40] Oetting A., Yen P.M. (2007). New insights into thyroid hormone action. Best Pract. Res. Clin. Endocrinol. Metab..

[41] Doležal P., Dvořáček J., Doležal J., Čermáková J., Zeman1 L., Szwedzia K. (2011). Effect of feeding yeast culture on ruminal fermentation and blood indicators of Holstein dairy cows. Acta Vet. Brno.

